# Development and Validation of Prediction Models for Hypertensive Nephropathy, the PANDORA Study

**DOI:** 10.3389/fcvm.2022.794768

**Published:** 2022-03-10

**Authors:** Xiaoli Yang, Bingqing Zhou, Li Zhou, Liufu Cui, Jing Zeng, Shuo Wang, Weibin Shi, Ye Zhang, Xiaoli Luo, Chunmei Xu, Yuanzheng Xue, Hao Chen, Shuohua Chen, Guodong Wang, Li Guo, Pedro A. Jose, Christopher S. Wilcox, Shouling Wu, Gengze Wu, Chunyu Zeng

**Affiliations:** ^1^Department of Cardiology, Daping Hospital, Third Military Medical University, Chongqing, China; ^2^Chongqing Key Laboratory for Hypertension Research, Chongqing Cardiovascular Clinical Research Center, Chongqing Institute of Cardiology, Chongqing, China; ^3^Department of Epidemiology, School of Public Health and Management, Chongqing Medical University, Chongqing, China; ^4^Department of Cardiology, Kailuan General Hospital, Tangshan, China; ^5^Department of Endocrinology, Southwest Hospital, Third Military Medical University, Chongqing, China; ^6^Division of Renal Disease & Hypertension, The George Washington University School of Medicine & Health Sciences, Washington, DC, United States; ^7^Division of Nephrology and Hypertension, Department of Medicine and Center for Hypertension, Kidney and Vascular Health, Georgetown University, Washington, DC, United States; ^8^State Key Laboratory of Trauma, Burns and Combined Injury, Daping Hospital, The Third Military Medical University, Chongqing, China; ^9^Cardiovascular Research Center of Chongqing College, Chinese Academy of Sciences, University of Chinese Academy of Sciences, Chongqing, China

**Keywords:** hypertension, hypertensive nephropathy, pulse pressure, chronic kidney disease, risk model

## Abstract

**Importance:**

Hypertension is a leading cause of end-stage renal disease (ESRD), but currently, those at risk are poorly identified.

**Objective:**

To develop and validate a prediction model for the development of hypertensive nephropathy (HN).

**Design, Setting, and Participants:**

Individual data of cohorts of hypertensive patients from Kailuan, China served to derive and validate a multivariable prediction model of HN from 12, 656 individuals enrolled from January 2006 to August 2007, with a median follow-up of 6.5 years. The developed model was subsequently tested in both derivation and external validation cohorts.

**Variables:**

Demographics, physical examination, laboratory, and comorbidity variables.

**Main Outcomes and Measures:**

Hypertensive nephropathy was defined as hypertension with an estimated glomerular filtration rate (eGFR) < 60 ml/min/1.73 m^2^ and/or proteinuria.

**Results:**

About 8.5% of patients in the derivation cohort developed HN after a median follow-up of 6.5 years that was similar in the validation cohort. Eight variables in the derivation cohort were found to contribute to the risk of HN: salt intake, diabetes mellitus, stroke, serum low-density lipoprotein, pulse pressure, age, hypertension duration, and serum uric acid. The discrimination by concordance statistics (C-statistics) was 0.785 (IQR, 0.770-0.800); the calibration slope was 1.129, the intercept was –0.117; and the overall accuracy by adjusted *R*^2^ was 0.998 with similar results in the validation cohort. A simple points scale developed from these data (0, low to 40, high) detected a low morbidity of 7% in the low-risk group (0–10 points) compared with >40% in the high-risk group (>20 points).

**Conclusions and Relevance:**

A prediction model of HN over 8 years had high discrimination and calibration, but this model requires prospective evaluation in other cohorts, to confirm its potential to improve patient care.

## Introduction

Hypertensive nephropathy (HN) is an independent risk factor for cardiovascular and cerebrovascular events (1). It is currently the second commonest cause of end- stage renal disease (ESRD) in the United States and third in Japan (2, 3). It accounts for 12% of new ESRD patients in Europe and 7% in China (4). Although inhibition of the renin-angiotensin-aldosterone-system may slow the progression of HN, most continue to have a decrease in GFR, despite good control of blood pressure (BP) (5). A lower BP may have some benefit in those with more proteinuria or obesity (6). However, a lower BP goal did not slow down the decrease in GFR in the African American Study of Kidney Disease and Hypertension (AASK) trial (7). Moreover, currently there is no treatment strategy to reverse HN once it is established or detect those who will develop HN.

Early diagnostic markers of HN include urinary albumin (8), serum cystatin C (9), and urinary N-acetyl-beta-glucosaminidase (10), but once present or abnormal levels are detected, irreversible damage to the kidney may have already occurred, despite aggressive control of BP (5, 11). Proteomics and genomics tests are expensive and have not clearly identified markers of HN (12–14). Therefore, we tested the hypothesis that a selection of readily available information, including demographics and simple clinical variables could be used to predict the development of HN, similar to a predictor of cardiovascular events (15). We developed a prediction model of HN and tested its 8-year occurrence. The model incorporated medical data from a large, community-based hypertensive population in China. We identified a high-risk population of patients who may benefit from early intervention if this becomes available, as recommended (16).

## Materials and Methods

### Study Population

#### Derivation Cohort

A derivation cohort was developed from the Kailuan Study data. This was a prospective, population-based cohort, representative of the real world. The participants consisted of currently employed and retired workers in the Kailuan Corporation. These subjects underwent health examinations in 2-year cycles. Participants examined from January 2006 to August 2007 with the diagnosis of hypertension were recruited and followed for eight years until December 2014.

#### Validation Cohort

An external validation cohort included hypertensive patients, who received medical care annually from June 2008 to December 2009 in community medical facilities of Hebei Province and Chongqing City, and followed up until December 2017. These patients were long-term residents of the community and received regular care at the community medical facilities, including free health checkup (Supplementary Figure 1).

The study was approved by the ethics Committee of Kailuan General Hospital and Daping Hospital and followed the Helsinki Declaration’s guidelines. All participants provided written informed consent.

**FIGURE 1:**
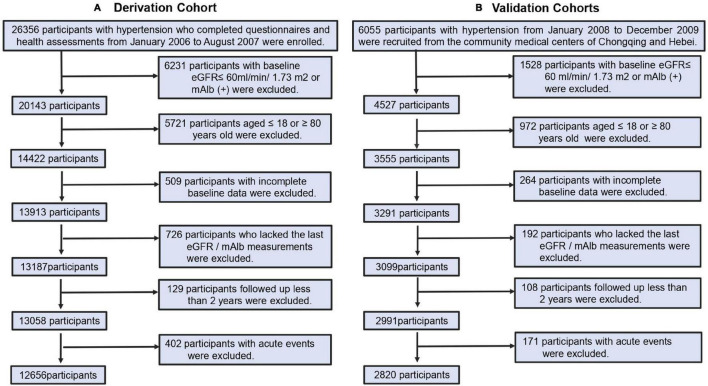
Cohort identification. **(A)** Process for the selection of participants in derivation cohorts. **(B)** Process for the selection of participants in external validation cohorts.

We enrolled participants between 18 and 80 years of age with a diagnosis of essential hypertension, according to the 2004 Chinese Guidelines for the Management of Hypertension, i.e., clinic systolic blood pressure (SBP) ≥ 140mmHg and/or diastolic blood pressure (DBP) ≥ 90mmHg, determined at three separate visits or currently taking antihypertensive medications for >1 year. Participants with incomplete data of candidate predictors, and those with renal dysfunction were excluded from the cohorts, as wells as those who developed sudden acute events. Renal dysfunction was defined as estimated glomerular filtration rate (eGFR) < 60 ml/min/1.73 m^2^ and/or proteinuria was diagnosed by urine dipstick (H12-MA, DIRUIN-600) with ≥1+, taken as a positive value. The eGFR was calculated from a modified version of the Modification of Diet in Renal Disease (MDRD) equation (17), adapted for Chinese patients with chronic kidney disease (CKD). The selection of participants in the cohorts is shown in Figure 1.

### Outcomes

The primary outcome was the occurrence of HN, defined as two eGFR 90 days apart <60 ml/min/1.73 m^2^ with hypertension.

### Candidate variables

The selection of study variables was obtained from systematic evaluation of 16 prospective and 2 retrospective study cohorts that enrolled 369,626 participants (18–35) and searched through Embase, PubMed, and the Cochrane Library database, until June 2020. This yielded 12 variables including sex, age, body mass index (BMI), DBP, SBP, antihypertensive medications, serum low-density lipoprotein (LDL), serum uric acid (UA), salt intake, fasting plasma glucose (FBG), diabetes mellitus (DM), and duration of the diagnosis of hypertension (Supplementary Figure 2).

The 2013 KHA-CARI guideline and other guidelines all state that smoking, history of cardiovascular disease [including myocardial infarction (MI)], and cerebrovascular disease (including stroke) should be used as screening factors for early CKD (36–40). Arterial pulse pressure (PP) is reported to be closely related to small artery atherosclerosis (41, 42), and an important risk factor for eGFR decline and development of CKD that is a better predictor of adverse renal outcomes than DBP and SBP (41, 42). Therefore, PP was also included as a candidate variable. Accordingly, a predictive model with 16 risk factors was evaluated. Questionnaires and clinical encounters were interrogated to obtain data, including sex, age, history of antihypertensive medications, DM, stroke, and MI by trained research doctors. Self-report was used to classify smoking status as “yes” or “no.” The salt (NaCl) intake “high” was defined as more than 10 g/day, “moderate” (6–<10 g/day), and “low” (<6 g/day). BP was measured by trained physicians and nurses. Weight, height, and BMI were measured during the interview. Blood samples were collected at the laboratories of Kailuan General Hospital, and Hebei and Chongqing community medical facilities.

### Statistics

#### Model Development

This used statistical criteria that followed stepwise selection and shrinkage techniques. First, univariable hazard ratios (HRs) were generated for all candidate predictor variables and evaluated, using the Cox proportional hazards model. Those with *p*-values < 0.05 were included directly in the analysis, and >0.05, <0.2 were considered clinically significant and were included in the initial selection. The least absolute shrinkage and selection operator (LASSO) (43, 44) method was also used to a backward selection of variables, in combination with a penalty on the absolute value of the regression coefficients; some were set to zero and others were shrunk. Selection was judged from the lambda with 1 SE of the minimum partial likelihood deviance. Nine variables were retained: age, DM, hypertension duration, salt intake, stroke, LDL, PP, SBP, and UA. Thereafter, multicollinearity was assessed from the variance inflation factor (VIF), that was defined as the inverse of tolerance. A VIF > 5.0 was considered an indication of harmful multicollinearity (45). Finally, the selected variables were assigned point scores, according to their β-regression coefficients from the adjusted multivariant regression in the risk model by adding the points corresponding to the risk factors, to define low-, moderate-, and high-risk groups. A nomogram and score sheet were also constructed to generate predicted morbidity of HN in 8 years for hypertensive patients. The estimated probability of HN from hypertension within eight years was equated to 1 – P_0_^exp (∑β *X*–∑β)^.

#### Model Performance

The performance of the prediction model was assessed by of discrimination, calibration, and overall predictive accuracy (46). Discrimination was from C-statistics that judges the model’s ability to distinguish two classes of outcomes for randomly selected pairs (with or without HN and high or low HN risk) and C-index value ranged from 0.50 to 1.00. Calibration was assessed graphically by plots of observed versus predicted probabilities of the outcome. It assesses the ability to evaluate, correctly, the predicted possibility of the outcome versus the observed outcome. The overall predictive accuracy was assessed from the R square (R^2^) statistic that represents the number of independent variables and the sample size, using adjusted R^2^.

Internal validation evaluates the reproducibility of the model and prevents overfitting. 2,000 iterations from the derivation data were bootstrapped to test model performance and optimism. External validation was selected to assess the model’s transportability and generalizability from which metrics of discrimination calibration and overall predictive accuracy were calculated.

This model’s reporting followed the TRIPOD (Transparent Reporting of a Multivariable Prediction Model for Individual Prognosis or Diagnosis) statement (16). All analyses were performed using SPSS version 26.0 and R version 3.6.3. Statistical significance was determined using a 2-sided test with a threshold *P*-value < 0.05.

## Results

### Characteristics of the Cohorts

Overall, 12,656 participants with hypertension were selected from the first survey from January 2006 to August 2007 and enrolled in the derivation cohort, and 2,820 hypertensive patients from Hebei and Chongqing City from June 2008 to December 2009 were enrolled in the validation cohort (Figure 1). Baseline characteristics are shown in Supplementary Table 1. In the derivation cohort, the median age was 56 years. The majority of participants were male and one third were smokers. Most reported consuming a low-salt diet, likely in response to advise, provided by the company for more than 10 years. Participants were managed by Kailuan Medical Group. One third received free antihypertensive medications for BP control ≤160/100 mmHg. The median duration of hypertension was 3.7 years in the derivation cohort and 3.5 years in the validation cohort. The patients in both cohorts had comparable SBP, DBP, and PP, and prevalence of DM. The incidence of stroke and MI was slightly higher in the validation cohort than in the derivation cohort, which may be related to the fact that the validation cohort was from medical center clinics primarily aimed at community-based chronic disease management. The participants in the validation cohort had higher median LDL, FPG, and UA than those in the derivation cohort. After a median follow-up of 6.5 years in the derivation cohort and 7.8 years in the validation cohort, 1,080 participants (8.5%) in the derivation cohort and 256 (9.1%) participants in the validation cohort developed HN.

### Model Development

As stated in the method, the variables included age, sex, smoking, BMI, SBP, DBP, PP, LDL, FPG, UA, DM, MI, stroke, history of antihypertensive medication, salt intake, and hypertension duration. To show the relationship of those risk factors with HN, a univariate Cox analysis evaluated the potential contributions of each variable to HN (Supplementary Table 2). The univariate Cox analysis selected 12 risk factors (*P* < 0.05) that were predictive but excluded smoking, DBP, and FPG. The contribution of sex in the development of HN was predicted by marginal *P* value (*P* = 0.082) but was included because of its clinical value.

**FIGURE 2:**
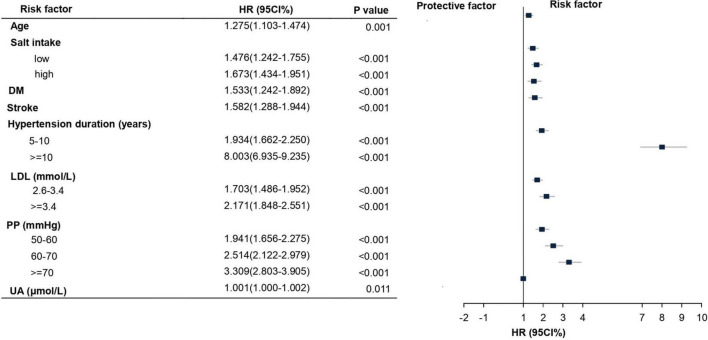
Multivariate Cox proportional hazard for the risk factors and their subgroups of HN. Hazard ratios (HR) and their corresponding 95% confidence intervals (CIs) for risk factors of HN development that are statistically significant. LDL, low-density lipoprotein; PP, pulse pressure; UA, uric acid.

**FIGURE 3:**
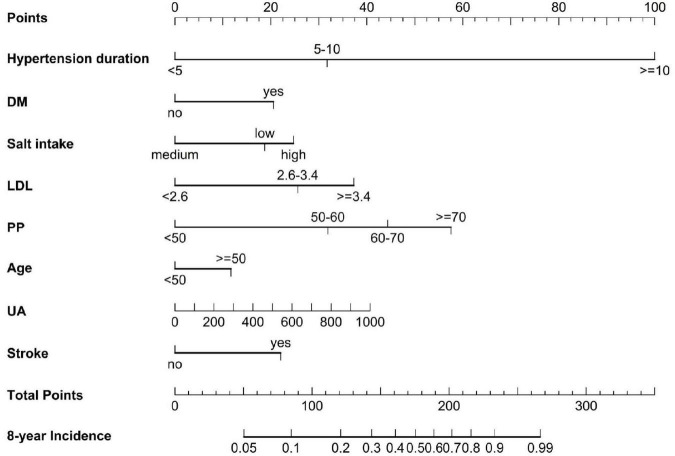
A nomogram to predict the 8-year incidence of HN. The line perpendicular from the corresponding axis of each risk factor determines the number of points received for each variable value. The sum of these numbers is located on the Total Points axis, and a line is drawn downward to the 8-year incident axes to determine the likelihood of 8-year HN risk. LDL, low-density lipoprotein, UA, uric acid.

The LASSO method was used to select further predictors and their regression coefficients for the model development to correct for overfitting, optimism, and miscalibration with the use of univariate Cox analysis, Supplementary Figure 3. Nine variables were selected: salt-intake, DM, stroke, serum LDL, UA, PP, age, hypertension duration, and SBP. The collinearity of one risk with other risk factors was assessed because one risk factor may affect the contribution of others risk factors; risk factors with collinearity >5 were excluded. We found that the collinearity between SBP and PP reached from 4.1 to 5.1; to avoid instability of regression coefficients, SBP was removed from the selected variables (Supplementary Table 3). The remaining eight risk factors were analyzed by multivariate COX proportional hazard analysis to yield hazard ratios (HR), as depicted in Figure 2. A nomogram model was developed from these results that depicted the contribution of each risk factor and the final estimated morbidity for each participant (Figure 3).

### Model Performance, Validation

**FIGURE 4:**
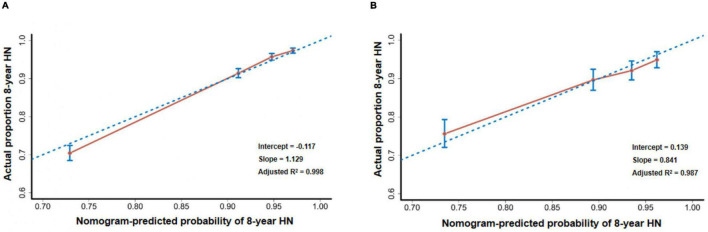
Calibration plots. **(A)** The calibration plot compares the predicted and actual HN probabilities at the eighth year of follow-up in internal validation. **(B)** The calibration plot compares the predicted and actual HN probabilities at the eighth year of follow-up in external validation.

The discrimination, calibration, and overall predictive accuracy in the derivation and validation cohorts of the model were bootstrapped from 2,000 iterations in the internal validation cohort. This yielded a C-statistics (the Harrell’s C-index) of 0.785 [95% confidence interval (CI), 0.770–0.800], while the calibration plots revealed good predictive accuracy of the nomogram with the slope of the curve of 1.129, an intercept of –0.117, and overall predictive accuracy by adjusted R^2^ of 0.998 (Figure 4A). The model’s predictive accuracy and stability in an external validation cohort yielded a similar C-statistics of 0.738 (95% CI, 0.705–0.771), calibration curves with similar slopes of 0.841, an intercept of 0.139, and an adjusted R^2^ of 0.987 (Figure 4B).

### Clinical Utility of the Prediction Model

**FIGURE 5:**
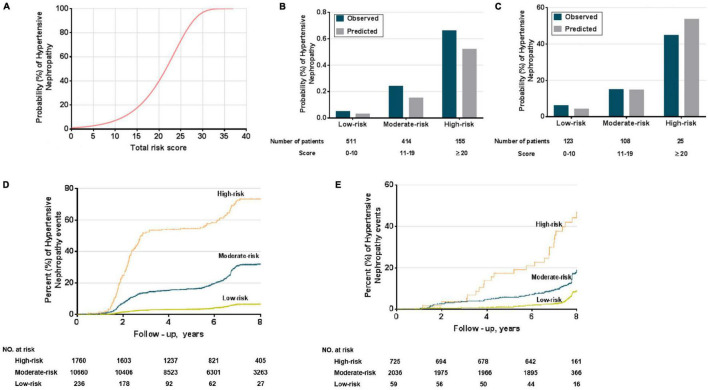
Performance of the HN risk-prediction model. **(A)** Probabilities (%) of HN, can be used to determine the individual’s corresponding predicted risk of developing HN. Comparison of observed and predicted onset rates of HN across risk groups in derivation **(B)** and external validation cohorts **(C)**; Kaplan–Meier survival curves for risk groups in derivation **(D)** and external validation cohorts **(E)**.

We converted the nomogram into a score sheet (Supplementary Table 4), using the estimated probability of HN over eight years as equal to 1 – P_0_^*exp* (∑β *X*–∑β)^. Each variable was given an individual probability of contributing to HN and the total risk score, based on individual probability, ranged from 0 (low) to 40 (high) (Figure 5A). Patients were classified as low-risk (0–10 points), moderate-risk (11–19 points), and high-risk (20 points or greater), based on the predicted 8-year incidence (<15%, 15–49%, ≥50%, respectively). The estimated risk scores for HN prediction in both derivation and validation cohorts were not different (Figures 5B,C, respectively). The value of the point score was tested from time-dependent development of HN in the risk groups. They segregated out the population into low-, moderate-, and high-risk of development of HN (Figures 5D,E).

## Discussion

A prediction model for the development of HN was based on the data from a large community study in China, using eight risk factors. The model has a good calibration, discrimination, and overall predictive accuracy, and potentially clinical decision-making value. Risk grouping from the derivation cohort was validated successfully in internal and external validation cohorts. Kaplan–Meier curves separated patients into moderate- and high-risk for developing kidney damage, thereby identifying a group that may require better monitoring, early intervention, or special care, we can intervene against some reversible risk factors such as PP, LDL, and UA, also we can protect the kidney using ACEI/ARB and statins as soon as possible. The risk score provided a simple means to distinguish moderate-risk and high-risk from low-risk patients. The application of the predictive model may provide a dynamic assessment of the risk of HN in hypertensive patients that would justify the continuous adjustment of their treatment strategies, as risk develops.

Controlling hypertension has had little impact on the development of CKD (5). The Multiple Risk Factor Intervention Trial (MRFIT) (28), as well as a large longitudinal cohort study (47), reported that ESRD remained prevalent in those with isolated systolic hypertension, despite a normal DBP and mean arterial pressure. The SPRINT subgroup of non-CKD patients reported that a DBP < 70 mmHg, paradoxically, was associated with a 30% increased risk of decreased eGFR (48). These results suggest that DBP may not be a strong predictor of adverse renal events. The Action in Diabetes and Vascular disease: preterAx and diamicroN-MR Controlled Evaluation (ADVANCE) study reported a 21% reduction in renal events with aggressive control of SBP (49) while the Action to Control Cardiovascular Risk in Diabetes-BP (ACCORD-BP) study reported a 16% reduction in microalbuminuria but had no impact on proteinuria or renal failure (50). However, the SPRINT subgroup analysis showed that acute kidney injury was three times greater in the intensive systolic treatment group than in the standard treatment group, but this could represent a hemodynamic, rather than a direct renal response to low BP (51). Thus, the use of SBP to predict HN may introduce bias. The early pathological changes of hypertension-related renal damage entail remodeling of small renal arteries that may be a response to a persistent increase in BP with elevated PP (52). Indeed, PP is an established marker of hypertensive target-organ damage whose correlation with damage was further confirmed in a Korean adult study (53) and the LOD-RISK study (54). Our model predicts that the ideal management of hypertension may require not only lowering SBP and DBP but also maintaining PP in a lower range to protect the kidneys from HN.

In comparison with previous CKD prediction models (55), the main difference is that we built a model for hypertensive patients and reflects the real-world situation. Lin et al. developed a prediction model for hypertensive renal damage (56), based on a small cross-sectional study of 582 hypertensive patients that was restricted to logistic regression analysis and required additional data. Our model was based on a prospective study of a cohort of 12,656 hypertensive patients followed for 8 years and validated in a separate cohort of 2,820 hypertensive patients. Our model only used variables readily available to clinicians from the Kailuan cohort which is a large-scale study in a Chinese community of more than 100,000 people/year, a long observation period, a relatively stable cohort population, which provides excellent conditions for population-based disease risk prediction modeling. We integrated traditional and non-traditional risk factors of CKD that were screened from literature reports and statistical methods to optimize risk factor selection. The prediction models were transformed into nomograms and risk scores, to provide a more convenient, and simple assessment for clinicians and patients. The risk stratification provides more clinically relevant information for medical guidance. We compared COX proportional stepwise regression with LASSO regression to filter variables to avoid overfitting the model and selected the Lambda value with the lowest cross-validation error to a more stable model. We followed the TRIPOD disease prediction model statement strictly, with excellent discrimination and calibration. Thus, we consider that our model would facilitate the earlier screening for the onset of HN and may contribute to HN prevention.

There are some limitations in our study. First, models were derived and validated in cohorts from Chongqing City and Hebei Province of China, which limits the generalization to other regions. Second, the median follow-up time was 8 years, but that follow-up time may be not sufficient to take fully into consideration the slow development of HN. Third, the study population in the derivation cohort was managed by one medical group that provided same, and free antihypertensive medication rather than individualized treatment. Fourth, some of the patients in our cohort accompany with diabetes, and in fact we could not identify very clearly whether the kidney damage was mainly from hypertension or diabetes.

In conclusion, a model for the development of HN, with high discrimination and calibration, was developed from routinely available variables in the clinic, predicted the long-term risk of kidney damage in hypertensive patients. The model performed very well in internal and external validation cohorts. Additional studies are needed to verify the clinical relevance of the model.

## Data Availability Statement

The original contributions presented in the study are included in the article/Supplementary Material, further inquiries can be directed to the corresponding authors.

## Ethics Statement

Written informed consent was obtained from the individual(s) for the publication of any potentially identifiable images or data included in this article.

## Author Contributions

SLW, GZW, and CZ: conceptualization. XY and BZ: methodology. XY, BZ, LZ, LC, JZ, SW, WS, YZ, XL, and CX: investigation. LZ, HC, and YX: software. XY and GZW: writing—original draft. GZW, PJ, CW, and CZ: writing—review and editing. CZ and GZW: funding acquisition. SC, GDW, and SLW: resources. CZ: project administration. GZW and CZ: supervision. All authors contributed to the article and approved the submitted version.

## Conflict of Interest

The authors declare that the research was conducted in the absence of any commercial or financial relationships that could be construed as a potential conflict of interest.

## Publisher’s Note

All claims expressed in this article are solely those of the authors and do not necessarily represent those of their affiliated organizations, or those of the publisher, the editors and the reviewers. Any product that may be evaluated in this article, or claim that may be made by its manufacturer, is not guaranteed or endorsed by the publisher.
