# Supplementary material for: Development and Validation of Prediction Models for Hypertensive Nephropathy, the PANDORA Study
Source: Front Cardiovasc Med. 2022 Mar 10;9:794768. doi: 10.3389/fcvm.2022.794768 (PMC8960139; doi:10.3389/fcvm.2022.794768)
Supplement: Supplementary file 1 [file Data_Sheet_1.doc]

**On-line Materials**

**eFigures:**

**eFigure 1:** Timeline of the study.

**eFigure 2:** Flow diagram outlining the literature search and study selection for risk factors of HN.

**eFigure 3:** Selection of variables, using the least absolute shrinkage and selection operator (LASSO).

**eTables:**

**eTable 1:** Baseline Characteristics of the Derivation and Validation Cohorts.

**eTable 2:** Univariate COX analysis of risk score for hypertensive nephropathy (HN).

**eTable 3:** Collinearity diagnosis of the nine-variable model with VIF value.

**eTable 4:** The risk score for hypertensive nephropathy (HN).

**1**

**
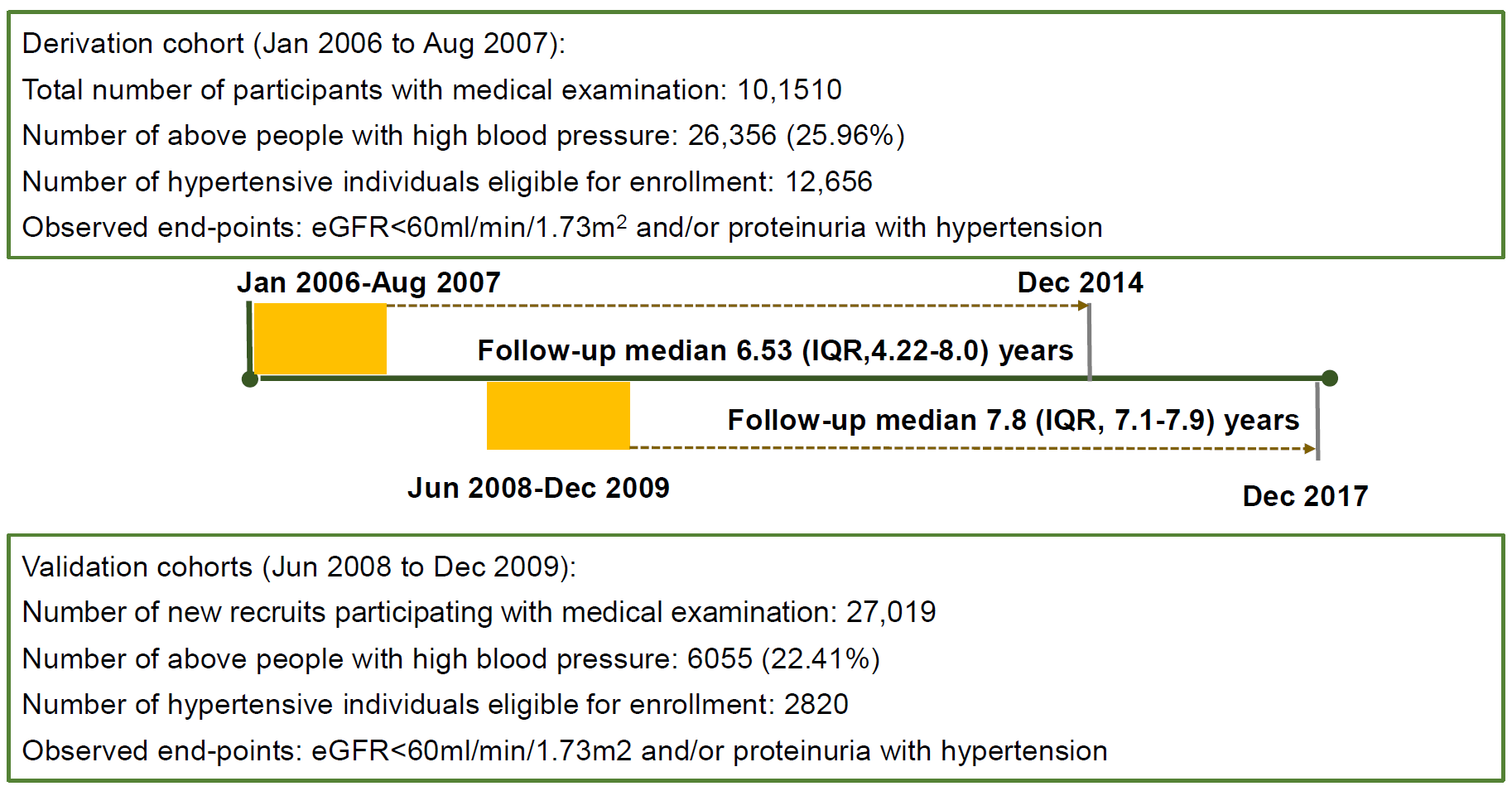
**

**eFigure 1.** Timeline of the study.

**2**

**
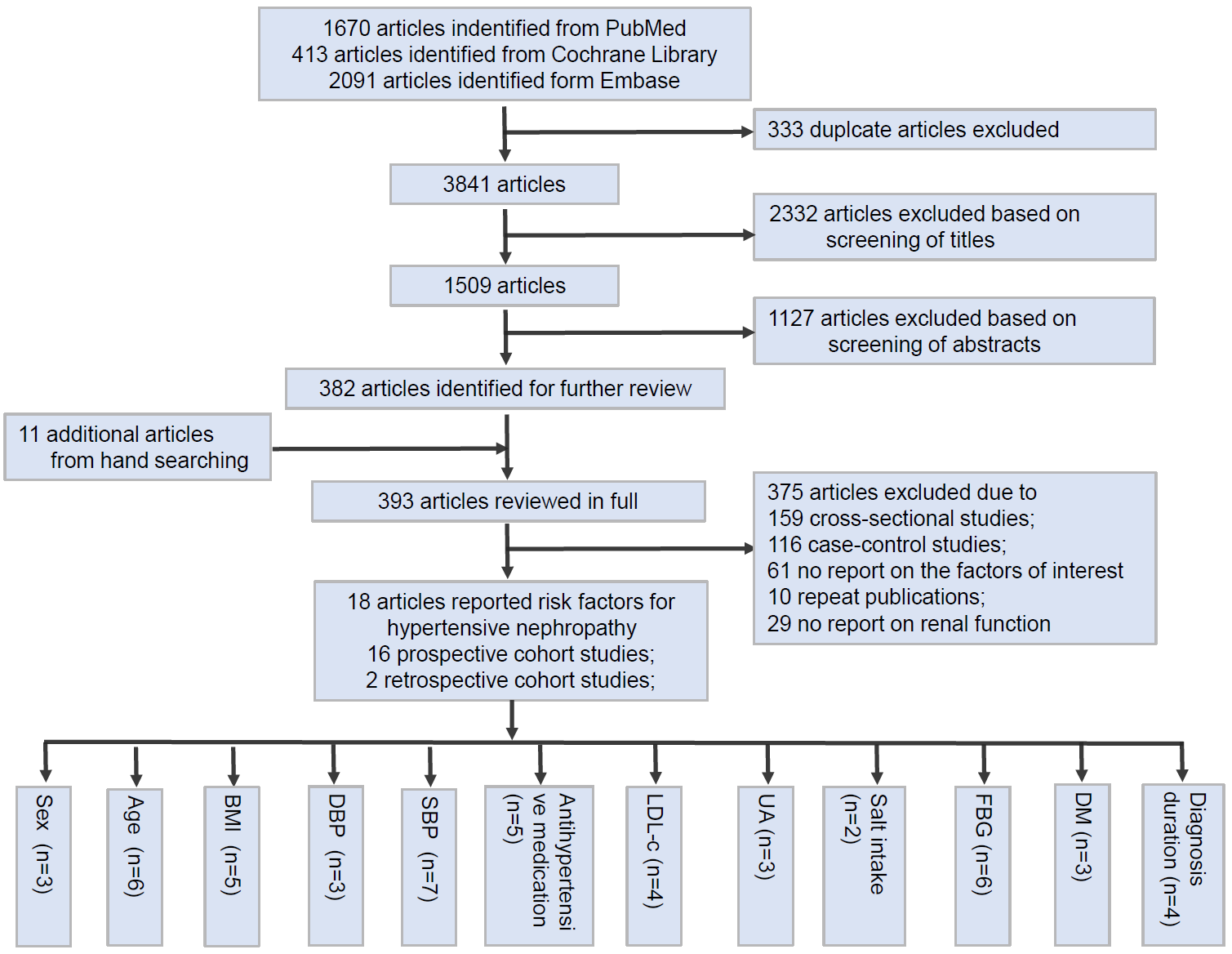
**

**eFigure 2.** Flow diagram outlining the literature search and study selection for risk factors of HN development in patients with hypertension.

**eFigure 3.** Variable selection by LASSO Cox proportional hazards model. A coefficient profile plot was produced against the log(λ) sequence (A). Nine variables with nonzero coefficients were selected by optimal lambda. By verifying the optimal parameter (λ) in the LASSO model, the partial likelihood deviance (binomial deviance) curve was plotted versus log(λ) and dotted vertical lines were drawn based on 1 standard error criteria (B).

**
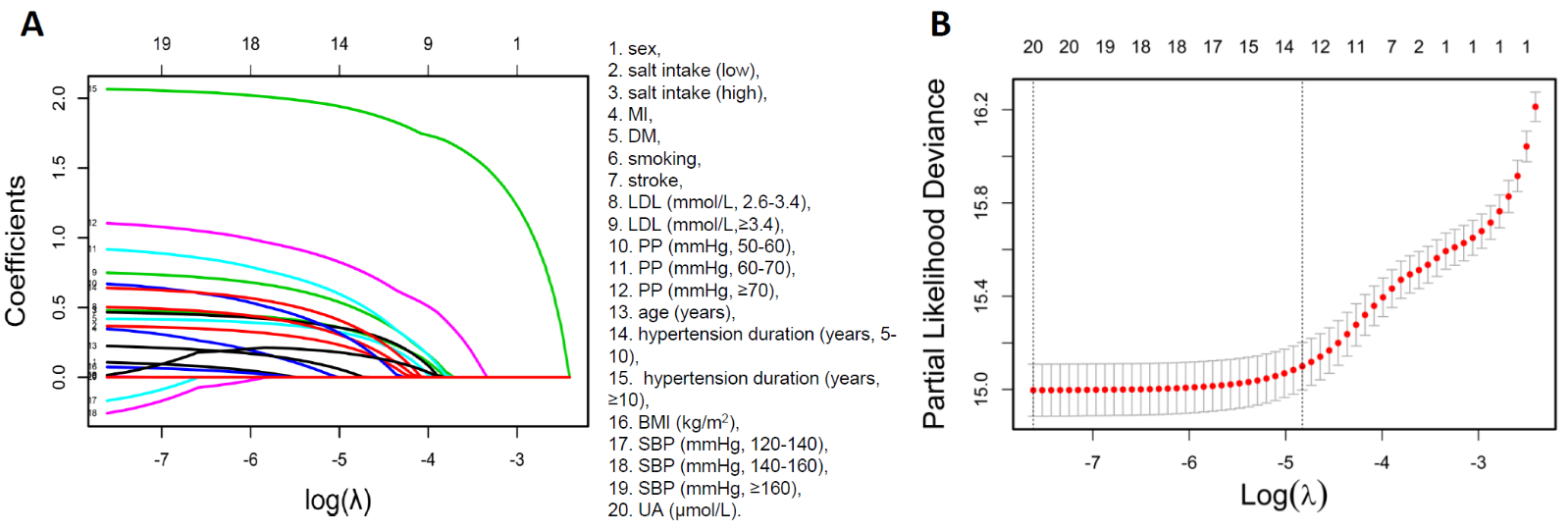
**

**4**

**eTable 1. Baseline Characteristics of the Derivation and Validation Cohorts**

| **Variables** | **Derivation Cohort**  **(n=12,656)** | **Validation Cohorts**  **(n = 2820)** |
| --- | --- | --- |
| **Demographics** | | |
| Age, median (IQR), y | 56 (48-63) | 55 (50-61) |
| Aged<50 y, No. (%) | 3536 (27.9) | 683 (24.2) |
| Aged≥50 y, No. (%) | 9120 (72.1) | 2137 (75.8) |
| Male sex, No. (%) | 9985 (78.9) | 2243 (79.5) |
| Smoking, No. (%) | 4372 (34.5) | 1102 (39.1) |
| Salt intake, No. (%) |  |  |
| Low | 9917 (78.4) | 2208 (78.2) |
| High | 1228 (9.7) | 275 (9.8) |
| Medium | 1151 (11.9) | 337 (12.0) |
| Hypertension duration, median (IQR), y | 3.7 (2.1-6.4) | 3.5 (1.9-6.2) |
| Tertile, No. (%) |  |  |
| <5 | 8161 (64.5) | 1864 (66.1) |
| 5 to 10 | 3524 (27.8) | 730 (25.9) |
| ≥10 | 971 (7.7) | 226 (8.0) |
| Antihypertensive medications, No. (%) | 3546 (28.0) | 1083 (38.4) |
| **Physical examination** | | |
| SBP, mean (SD), mm Hg | 145 (18) | 144 (19) |
| DBP, mean (SD), mm Hg | 91 (10) | 93 (12) |
| PP,mean (SD), mm Hg | 50 (15) | 51 (14) |
| Tertile, No. (%) |  |  |
| <50 | 7548 (59.6) | 1387 (49.2) |
| 50 to 60 | 2512 (19.8) | 622 (22.1) |
| 60 to 70 | 1423 (11.2) | 454 (16.1) |
| ≥70 | 1173 (9.3) | 357 (12.6) |
| BMI, mean (SD), kg/m^2^ | 25.8 (3.4) | 26.1 (3.3) |
| **Comorbid conditions** | | |
| DM, No. (%) | 529 (4.2) | 121 (4.3) |
| Stroke, No. (%) | 514 (4.1) | 220 (7.8) |
| MI, No. (%) | 1147 (4.2) | 188 (6.7) |
| **Laboratory data** | | |
| LDL, mean (SD) mmol/L | 2.4 (1.0) | 2.5 (0.8) |
| Tertile, No. (%) |  |  |
| <2.6 | 7981 (63.1) | 1532 (54.3) |
| 2.6 to 3.4 | 3215 (25.4) | 854 (30.3) |
| ≥3.4 | 1460 (11.6) | 434 (15.4) |
| FPG, mean (SD), mmol/L | 5.6 (1.7) | 6.0 (1.7) |
| UA, mean (SD), mmol/L | 300 (87) | 308 (87) |
| Tertile, No. (%) |  |  |
| <360 | 9852 (77.8) | 2097 (74.3) |
| 360 to 540 | 2671 (21.1) | 687 (24.4) |
| ≥540 | 133 (1.1) | 36 (1.3) |
| **Outcome** | | |
| Follow-up, median (IQR), y | 6.5 (4.2-8.0) | 7.8 (7.1-7.9) |
| Hypertensive nephropathy events, No. (%) | 1080 (8.5) | 256 (9.1) |

**5**

Abbreviations: IQR, interquartile range; BMI, body mass index; SBP, systolic blood pressure; DBP, diastolic blood pressure; PP, pulse pressure; DM, diabetes mellitus; MI, myocardial infraction; LDL, low density lipoprotein; FPG, fasting plasma glucose; UA, uric acid.

**6**

Abbreviations: BMI, body mass index; MI, myocardial infraction; DM, diabetes mellitus; FPG, fasting plasma glucose; SBP, systolic blood pressure; DBP, diastolic blood pressure; PP, pulse pressure; LDL, low density lipoprotein; UA, uric acid. **7**

| Risk factors | | β | HR (95%CI) | SE | Z-statistics | P Value |
| --- | --- | --- | --- | --- | --- | --- |
| Sex | Female | - | - | - | - |  |
|  | Male | 0.14 | 1.14 (0.98 to 1.33) | 0.07 | 1.738 | 0.08 |
| Age (years) | <50 | - | - | - | - |  |
|  | ≥50 | 0.20 | 1.22 (1.06 to 1.00) | 0.07 | 2.76 | 0.005 |
| Salt intake | Medium | - | - | - | - |  |
|  | High | 0.83 | 2.29 (1.62 to 2.29) | 0.07 | 10.80 | <0.001 |
|  | Low | 0.65 | 1.09 (1.97 to 2.66) | 0.08 | 7.56 | <0.001 |
| Smoking | No | - | - | - | - |  |
|  | Yes | 0.15 | 1.16 (1.03 to 1.32) | 0.06 | 2.49 | <0.013 |
| Antihypertensive medications | yes | -0.11 | 1.11 (0.97 to 1.27) | 0.06 | 2.51 | 0.21 |
|  | no |  |  |  |  |  |
| BMI (kg/m^2^) | <24 | - | - | - | - |  |
|  | ≥24 | 0.18 | 1.20 (1.04 to 1.37) | 0.06 | 2.65 | 0.008 |
| MI | No | - | - | - | - |  |
|  | Yes | 0.63 | 1.87 (1.42 to 2.47) | 0.14 | 4.51 | <0.001 |
| Stroke | No | - | - | - | - |  |
|  | Yes | 0.93 | 2.55 (2.08 to 3.11) | 0.10 | 9.15 | <0.001 |
| DM | No | - | - | - | - |  |
|  | Yes | 0.91 | 2.50 (2.03 to 3.07) | 0.10 | 8.73 | <0.001 |
| Hypertension duration (years) | <5 | - | - | - | - |  |
|  | 5-10 | 0.73 | 2.08 (1.79 to 2.42) | 0.07 | 9.55 | <0.001 |
|  | ≥10 | 2.29 | 9.87 (8,57 to 11.35) | 0.07 | 31.98 | <0.001 |
| FPG (mmol/L) | <6.1 | - | - | - | - |  |
|  | 6.1-7.0 | 0.08 | 1.08 (0.89 to 1.32) | 0.10 | 0.80 | 0.42 |
|  | ≥7.0 | 0.17 | 1.19 (0.99 to 1.44) | 0.09 | 1.88 | 0.05 |
| SBP (mmHg) | <120 | - | - | - | - |  |
|  | 120-140 | -0.001 | 0.99 (0.76 to 1.31) | 0.13 | -0.01 | 0.99 |
|  | 140-160 | 0.35 | 1.42 (1.09 to 1.84) | 0.13 | 2.60 | 0.009 |
|  | ≥160 | 0.90 | 2.47 (1.88 to 3.24) | 0.13 | 6.51 | <0.001 |
| DBP (mmHg) | <80 | - | - | - | - |  |
|  | 80-90 | -0.05 | 0.94 (0.79 to 1.12) | 0.08 | -0.62 | 0.53 |
|  | 90-100 | 0.09 | 1.09 (0.92 to 1.30) | 0.08 | 1.03 | 0.30 |
|  | 100-110 | 0.03 | 1.31 (0.81 to 1.31) | 0.12 | 0.02 | 0.78 |
|  | ≥110 | 0.28 | 1.32 (0.97 to 1.81) | 0.16 | 1.77 | 0.07 |
| PP (mmHg) | <50 | - | - | - | - |  |
|  | 50-60 | 0.71 | 2.03 (1.73 to 2.38) | 0.08 | 8.78 | <0.001 |
|  | 60-70 | 1.11 | 3.02 (2.56 to 3.58) | 0.08 | 12.93 | <0.001 |
|  | ≥70 | 1.42 | 4.15 (3.52 to 4.89) | 0.08 | 17.10 | <0.001 |
| LDL (mmol/L) | <2.6 | - | - | - | - |  |
|  | 2.6-3.4 | 0.56 | 1.75 (1.53 to 2.01) | 0.06 | 8.12 | <0.001 |
|  | ≥3.4 | 0.89 | 2.44 (2.08 to 2.86) | 0.08 | 10.98 | <0.001 |
| UA (μmol/L) |  | 0.002 | 1.001 (1.001 to 1.003) | 0.0003 | 5.94 | <0.001 |

**eTable 2. Univariate COX analysis of risk score for hypertensive nephropathy (HN)**

**eTable 3. Collinearity diagnosis of the nine-variable model with VIF value**

| **Variables** | | **VIF** |
| --- | --- | --- |
| Hypertension duration (years) | 5-10 | 1.26 |
|  | ≥10 | 1.30 |
| DM |  | 1.04 |
| Stroke |  | 1.05 |
| Salt intake | low | 1.07 |
|  | high | 1.09 |
| SBP (mm Hg) | 120-140 | 4.09 |
|  | 140-160 | 5.10 |
|  | ≥160 | 4.68 |
| LDL (mmol/L) | 2.6-3.4 | 1.14 |
|  | ≥3.4 | 1.16 |
| PP (mm Hg) | 50-60 | 1.36 |
|  | 60-70 | 1.40 |
|  | ≥70 | 1.64 |
| Age (years) |  | 1.03 |
| UA (μmol/L) |  | 1.03 |

Abbreviations: VIF, variance inflation factor; DM, diabetes mellitus; SBP, systolic blood pressure; LDL, Low density lipoprotein; PP, pulse pressure; UA, uric acid.

**8**

**eTable 4. The risk score for hypertensive nephropathy (HN)**

| **Risk factor for HN** | **Category** | **Risk score** |
| --- | --- | --- |
| **Salt intake** | Medium low high |  |
|  | 0 2 3 |  |
| **Stroke** | No Yes |  |
|  | 0 2 |  |
| **LDL (mmol/L)** | <2.6 2.6-3.4 ≥3.4 |  |
|  | 0 3 4 |  |
| **PP (mm Hg)** | <50 50-60 60-70 ≥70 |  |
|  | 0 3 5 6 |  |
| **Age (years)** | <50 ≥50 |  |
|  | 0 1 |  |
| **Hypertension duration (years)** | <5 5-10 ≥10 |  |
|  | 0 3 11 |  |
| **DM** | No yes |  |
|  | 0 2 |  |
| **UA (mmol/L)** | <360 360-540 ≥540 |  |
|  | 0 1 2 |  |
| Total risk score= | |  |

Abbreviations: LDL, Low density lipoprotein; PP, Pulse pressure, DM, Diabetes, UA, Uric acid.

**9**
